# Evaluation of an Oral Supplemental Cannabidiol Product for Acceptability and Performance in Mature Horses

**DOI:** 10.3390/ani13020245

**Published:** 2023-01-10

**Authors:** Julia M. Leise, Jessica L. Leatherwood, Brittany L. Paris, Kelly W. Walter, James M. George, Rafael E. Martinez, Kati P. Glass, Chih-Ping Lo, Travis P. Mays, Tryon A. Wickersham

**Affiliations:** 1 Department of Animal Science, Texas A&M University, College Station, TX 77843, USA; 2 Agricultural Science Department, Truman State University, Kirksville, MO 63501, USA; 3 School of Agricultural Sciences, Sam Houston State University, Huntsville, TX 77340, USA; 4 Veterinary Large Animal Clinical Sciences, Texas A&M University, College Station, TX 77843, USA; 5 Texas Veterinary Medical Diagnostic Laboratory, TVMDL, College Station, TX 77843, USA

**Keywords:** cannabidiol, equine, blood chemistry

## Abstract

**Simple Summary:**

Cannabidiol, better known as simply CBD, is one of the non-psychoactive compounds found in hemp. Its supplementation to horses is gaining in popularity as a potential alternative to conventional pharmaceutical treatments for a variety of conditions such as arthritis and anxiety. Despite potential advantages of CBD supplementation, it may have a negative impact on equine health and performance. More scientific research is needed to form recommendations for its use in horses; therefore, this study was designed to investigate supplementation of CBD oil over 28 days in 30 horses. Targeted levels of supplementation were to provide three levels for comparison (0.0 mg/kg, 0.75 mg/kg, or 1.50 mg/kg); however, the supplements collected during the study were analyzed to be substantially lower than targeted levels when tested at a verified testing facility (0.12 mg/kg and 0.13 mg/kg). At this level of supplementation, CBD oil was well-accepted, CBD was not detectible in blood samples, and blood chemistry parameters to assess liver and kidney function were not adversely affected as a result of supplementation. However, more research is needed to describe the discrepancy between formulated levels compared to tested levels of CBD supplements, in order to make recommendations for its application in the horse industry.

**Abstract:**

Thirty stock type geldings (15 ± 3 years; 556 ± 63 kg BW) were used in a randomized complete design over 28 days to determine the influence of cannabidiol (CBD) oil supplementation levels on body weight, body condition, and blood chemistry. Horses were randomly assigned to one of three dietary treatments (n = 10 per treatment) formulated with canola oil to provide 1.50 mg CBD/kg BW (TRTA), 0.75 mg CBD/kg BW (TRTB), or 0.00 mg CBD/kg BW (canola oil; CTRL). Treatments were top-dressed onto concentrate and individually administered twice daily. Horses were maintained in adjacent dry lots and received coastal bermudagrass hay ad libitum. Body weight and body condition scores (BCS) were obtained every 14 days. On day 0 and 28, blood was collected via jugular venipuncture and serum was harvested to perform a blood chemistry panel and drugs of abuse screening at the Texas Veterinary Medical Diagnostic Laboratory. Data were analyzed using PROC MIXED of SAS (v9.4), and the model included treatment, time, and the treatment × time interaction, and linear and quadratic orthogonal polynomial contrasts to partition sum of squares. Analysis of composited treatment samples revealed lower CBD concentrations than indicated from initial testing by the manufacturer (0.13 mg CBD/kg in TRTA; 0.12 mg CBD/kg in TRTB). At this level of supplementation, canola-based CBD oil was well-accepted by mature horses, banned substances were not detectable in blood, and blood chemistry parameters were not adversely affected as a result of supplementation. More research is warranted to describe the discrepancy between formulated levels compared to tested levels of CBD in the canola-based supplement.

## 1. Introduction

The use of cannabidiol (CBD), one of the non-psychoactive compounds found in hemp, is gaining in popularity as a potential alternative to conventional pharmaceutical treatments for a variety of conditions, including but not limited to arthritis and anxiety. Studies conducted in mice and horses have shown that CBD reduces the production of inflammatory cytokines such as tumor necrosis factor-α (TNFα), which may help reduce inflammation in various body tissues or may have a calming effect and lessen anxiety in horses [1,2]. Horses receiving 40 g of a pelleted CBD supplement containing 100 mg of CBD over 6 weeks were less reactive to a novel stimulus compared to a control group; however, heart rates were not different and blood serum was not evaluated [3].

Despite the potential advantages of CBD supplementation, dietary CBD may have a negative impact on equine health and performance. Adverse responses reported in mice provided a dose of 615 mg CBD/kg body weight (BW) were lethargy, loss of appetite, and body weight loss [4]. It has also been identified in multiple studies that CBD may induce the cytochrome p450 mediated oxidative metabolism in the liver and cause an increase in alkaline phosphatase (ALP) and alanine aminotransferase (ALT) activity, which suggests that liver injury is present. The increase in serum ALP levels were seen in dogs at 2 mg CBD/kg BW and at 2.5 mg CBD/kg BW [5,6]. Increases in ALT serum levels were reported in mice at 615 mg CBD/kg BW and in cats at 2 mg CBD/kg BW [4,7]. These studies indicate that CBD may have the potential for dose-dependent liver injury. Another useful indicator of long-term liver damage in horses is gamma-glutamyltransferase (GGT), due to its association with hepatocytes responsible for bile production [8,9].

In addition to the potential of liver injury, CBD may have a positive or negative effect on kidney function depending on the dosing level. Cannabinoid Type 1 (CBR1) and Type 2 (CBR2) receptors are found in the kidneys and interact with G-proteins, which produce several effects including an increase in calcium levels [10]. Elevated blood urea nitrogen (BUN), calcium (Ca), and creatinine are additional indicators of kidney function and signify if the kidneys are under stress [11]. Studies in horses have indicated similar concerns to health as those in rodents and companion animals. A pilot pharmacokinetics study evaluating three different dosage levels (50 mg, 100 mg, and 250 mg) of a pelleted CBD supplement given to horses in a single dose was performed by Draeger et al. [12]. Although serum chemistry was within normal ranges, BUN levels increased with CBD supplementation level and creatinine decreased then increased as supplementation level increased. A study utilizing 2.0 mg/kg was well tolerated (in terms of vital signs, appetite, and attitude) over a period of 7 days [13]. However, when supplemented over 6 weeks, another trial found supplementation at 0.5 mg/kg or 1.5 mg/kg resulted in mild hypocalcemia and elevated liver enzymes in all horses [14].

Previous work involving dietary supplementation levels has focused primarily on companion animals, including dogs and cats, with limited information available regarding appropriate CBD oil supplementation levels in mature horses. Previous research in horses utilized 100 mg over 6 weeks, and observed decreased reactivity [3], or a single dose of 50 mg, 100 mg, and 250 mg in a pelleted supplement, and observed increasing levels of BUN indicating possible kidney stress [12]. A single oral dose (suspended in sesame oil) of 0.5, 1, or 2 mg/kg of CBD was utilized in exercising thoroughbreds [15] to evaluate potential anti-inflammatory effects, and was well tolerated by horses with no significant behavioral or gastrointestinal abnormalities observed.

Therefore, the objectives of the current study were to (1) determine the influence of CBD oil supplementation levels on the health of mature horses over 28 days by monitoring BW, BCS and grain intake, (2) evaluate factors associated with kidney and liver health by conducting a blood chemistry profile to measure BUN, creatinine, total protein, albumin, Ca, P, glucose, and liver enzymes, and to (3) identify the presence of CBD along with other banned substances ((CBD, 7-carboxycannabidiol, 7-Nor-7-carboxycannabidiol, and tetrahydrocannabinol (THC)) in serum as a result of dietary CBD oil supplementation.

## 2. Materials and Methods

### 2.1. Horses and Diets

Thirty Quarter horse geldings (mean ± SD; 14.8 ± 2.6 years and initial body weight (BW) 556 ± 63 kg) were selected from a herd at Texas A&M University (College Station, TX, USA) and utilized in a completely randomized design to determine the effects of dietary CBD on mature horses. Horses were evenly stratified and balanced based on BW and age, and randomly assigned to one of three dietary treatment groups (n = 10 horses/treatment) for a 28-day trial. Before initiating the study, all horses were placed on a similar nutritional background for 30 days, and blood samples were obtained via jugular venipuncture into a 7.5 mL sterile non-additive collection tube (BD Vacutainer, BD#367987, Fisher Scientific, Pittsburgh, PA, USA). A blood chemistry profile panel was performed (Texas Veterinary Medical Diagnostic Laboratory, TVMDL, College Station, TX, USA) and evaluated by a board-certified veterinarian to ensure values were within normal physiological limits prior to enrolling horses into the study.

Horses meeting the inclusion criteria received dietary treatments consisting of canola oil supplementation (CTRL) and two levels of CBD containing oil (canola base; Arrowhead Laboratories, Costa Mesa, CA, USA) formulated by the manufacturer to provide 1.50 mg CBD/kg BW (TRTA) or 0.75 mg CBD/kg BW (TRTB) of CBD. Treatments were divided evenly between the two daily concentrate meals, top-dressed and thoroughly mixed immediately prior to feeding. Levels of CBD supplementation were determined based on the previous literature in horses [15] and other species, using up to 1.5 mg CBD/kg BW [5,7,16,17] to avoid negative implications on liver health. An oil-based formulation was chosen due to ease of administration and higher bioavailability when compared to solid-based formulations [5,18,19,20].

A commercially available concentrate (Producer’s Golden Years, Producer’s Coop, Bryan, TX, USA, Table 1) was offered (0.75% BW (as-fed)) daily to meet or slightly exceed the nutritional requirements for mature horses at maintenance [21]. Concentrate was provided at 12 h intervals using attachable feed bags (Derby Originals LLC; North Canton, OH, USA), and horses had 60 min to consume concentrate. Any remaining orts were weighed and resulting daily intakes were calculated.

Horses were group-housed and placed into adjacent dry lots with ad libitum access to water and coastal bermudagrass (*Cynodon dactylon*) hay in the form of round bales, and were allowed 2 h per day on the same coastal bermudagrass pasture (Table 1). Body weight was obtained every 14 days using a platform scale and body condition scores (BCS) were assigned independently by three observers [22]. Samples of grain, hay, and pasture were composited, and nutrient content was determined (Table 1) using a commercial laboratory (Cargill Inc., Elk River Forage Lab, Elk River, MN, USA). Composited samples of each oil (TRTA, TRTB, CTRL) were also obtained by collecting weekly samples of 20 mL. Levels of CBD in each oil composite sample were determined by an independent laboratory (TVMDL, College Station, TX, USA).

### 2.2. CBD Oil Extraction

A 20 μL sample was pipetted into a screw-top tube. After adding 5 mL of 0.1% acetic acid acetonitrile to the extract, the tube was capped, and roto-racked for 10 min and centrifuged for 5 min at 2700× *g*. The supernatant was then transferred to another tube and evaporated under nitrogen at approximately 40 °C, and was reconstituted with 1 mL of 20% methanol before loading onto the CEREX^®^ WWP cartridge. The target analyte was eluted into two fractions using 1 mL of Hexan and 1 mL of ethyl acetate/Hexan (1:1, *v*/*v*). The two fractions were then combined and dried under nitrogen at 40°C. The extract was reconstituted with 80 μL 50% acetonitrile for instrumental analysis. All LC–MS/MS analyses were performed using a Thermo Altis (Thermo, San Jose, CA, USA) triple quadrupole mass spectrometer with an electrospray ionization (ESI) source [23,24].

### 2.3. Sample Collection and Analysis

Blood samples were collected prior to the start of the trial (day 0) and on day 28 following the onset of supplementation. Serum was collected into non-additive sterile blood collection tubes and allowed to separate at room temperature for 1 h prior to centrifugation. Samples were centrifuged at 2700× *g* for 20 min (ALC, PM140R, Thermo Fisher Sci., Waltham, MA, USA), and then harvested and transported to TVMDL on ice to evaluate blood parameters and liver enzymes.

Serum samples were also tested for the panel of “drugs of abuse” using liquid chromatography/mass spectrometry at a commercial laboratory (TVMDL, College Station, TX, USA) for the detection of amphetamine, methamphetamine, 3,4-Methylenedioxymethamphetamine, bromazepam, demoxepam, diazepam, etizolam, flunitrazepam, lorazepam, midazolam, nordazepam, prazepam, temazepam, acepromazine, 2-(1-hydroxyethyl) promazine sulfoxide (2-HEPS), chlorpromazine, propionylpromazine, promethazine, allobarbital, aprobarbital, phenobarbital, pentobarbital, secobarbital, alfentanil, buprenorphine, butorphanol, codeine, fentanyl, heroin, hydrocodone, levorphanol, morphine, oxymorphone, cannabidiol (CBD), 7-carboxycannabidiol, 7-Nor-7-carboxycannabidiol, tetrahydrocannabinol (THC), delta9-THC, 11-nor-9-carboxy-THC, 11-hydroxy-delta9-THC, carboxy-delta9-THC glucuronide, AM-2201, JWH-122, JWH-200, JWH-210, JWH-081, JWH-019, JWH-203, JWH-250, JWH-015, HU-211, [(±)-CP 47, 497], [(±)-CP 47, 497 C8 Homologue], RCS-4, RCS-8, carisoprodol, cocaine, benzoylecgonine (BEG), GABA, lidocaine, hydroxylidocaine, ketamine, lysergic acid diethylamide, mephedrone, mephenesin, methylone, methylphenidate, naltrexone, pentazocine, phencyclidine, psilocin, psilocybin, pyrovalerone, sertraline, tramadol, trimeprazine, and zolpidem.

### 2.4. Statistical Analysis

Data were initially evaluated for normality and outliers were identified using box plots of the residuals and removed if greater than two standard deviations from the mean [25]. Investigators remained blinded to treatments throughout statistical analysis. Bottles were alphabetically coded (A, B, C) in pre-weighed bottles provided by the manufacturer.

Body weight and BCS were analyzed separately from values obtained from the blood chemistry panel. For performance variables, the model contained fixed effects for treatment, time and an interaction for treatment × time. Both RANDOM and REPEATED statements were also utilized in the model to account for variability between animals. A covariate was used to account for differences in blood chemistry on d 0, and the main effect tested was treatment. Linear and quadratic effects were tested in the form of orthogonal contrasts [26]. Contrast values were determined using PROC IML in SAS. Significance was declared at *p* ≤ 0.05 and a trend if *p* ≤ 0.10.

## 3. Results

### 3.1. Cannabidiol Concentrations

Levels of CBD supplementation were formulated by the manufacturer to achieve a targeted intake of 1.5 mg CBD/kg BW (TRTA) and 0.75 mg CBD/kg BW (TRTB) per day. The required concentrations to achieve these targets were 46 mg/mL (TRTA) and 23 mg/mL (TRTB). However, composited samples of each treatment that were obtained during the trial revealed supplemented products contained 4.0 mg/mL (TRTA) and 3.7 mg/ML (TRTB) when tested by an independent laboratory (TVMDL). Therefore, horses received an average of 0.13 mg CBD/kg BW on TRTA and 0.12 mg CBD/kg BW on TRTB. This represents a 91.3% and 84% reduction from targeted to actual intake of CBD on TRTA and TRTB in the current study.

### 3.2. Intake and Performance Characteristics

The current study examined the effects of cannabidiol oil on horse performance and blood chemistry parameters in mature horses. Top-dressing the canola-based oil onto the concentrate did not affect (*p* = 0.97) the intake of concentrate. Similarly, there was no effect of CBD oil supplementation on BW (*p* = 0.97) or BCS (*p* = 0.44); however, there was an influence of time, and BCS increased (*p* ≤ 0.01) in all horses (mean ± SEM; 6.04 ± 0.14 to 6.33 ± 0.14) to day 14 then decreased from day 14 to 28 (mean ± SEM; 6.13 ± 0.14). While differences were observed in this study over time, BCS remained within normal ranges of 4 to 6 for mature horses at maintenance [22,27].

### 3.3. Blood Chemistry Parameters, Enzyme Activity, and Drugs of Abuse Screening

A linear dose response was observed (*p* ≤ 0.05) for blood Ca levels, with Ca concentrations increasing from 11.37 ± 0.07 mg/dL in CTRL to 11.67 ± 0.07 mg/dL in TRTA horses (Table 2). Levels of serum Ca, following 28 d of supplementation, were within the physiologically normal reference range (11.2 to 13.0 mg/dL, TVMDL, College Station, TX). In contrast to Ca, creatinine levels tended to decrease (*p* = 0.06) with increasing levels of CBD supplementation (CTRL = 1.41 mg/dL, TRTB = 1.34 mg/dL, TRTA = 1.31 mg/dL (Table 2)). The remaining blood chemistry parameters, including blood urea nitrogen, total protein, albumin, total bilirubin, bilirubin direct, albumin:globulin, globulins, glucose, phosphorous, sodium, potassium, Na:K, and Cl, were not influenced (*p* ≥ 0.13) by dietary treatment.

Gamma-glutamyl transferase demonstrated a quadratic dosage response, as TRTB was greater than CTRL (*p* = 0.03; Table 3). The remaining liver enzymes (alkaline phosphatase, creatine kinase, aspartate aminotransferase, glutamate dehydrogenase) were not influenced by dietary treatment (*p* ≥ 0.16) in this trial.

No drugs of abuse (including, but not limited to CBD, 7-carboxycannabidiol, 7-Nor-7-carboxycannabidiol, and tetrahydrocannabinol (THC)) were detected in blood on day 0 and the following 28 days of supplementation in all horses, regardless of treatment.

## 4. Discussion

### 4.1. Cannabidiol Concentrations

Levels of CBD supplementation were formulated by the manufacturer to achieve a targeted intake of 1.5 mg CBD/kg BW (TRTA) and 0.75 mg CBD/kg BW (TRTB) per d. However, composited samples of each treatment that were obtained during the trial revealed supplemented products contained 0.13 mg CBD/kg BW (TRTA) and 0.12 mg CBD/kg BW (TRTB).

The literature indicates inconsistencies across the industry in concentrations due to plant type and processing [28]. This would not explain the present study results, as differences were seen in measured concentrations of the same product. Temperature and photo-instability have a role [29], but would not account for the magnitude of differences seen here. Supplements were maintained in a room temperature environment until needed and only kept in the barn (ambient outdoor temperatures) for one to three days in a shaded location.

Additionally, there is a lack of standardized testing methods for CBD products, which presents a significant limitation, emphasizing the need for the adoption of testing standards by industry, states, or even the federal government [30]. Note that the producer measured concentrations using High Performance Liquid Chromatography (HPLC) and the verification lab (TVMDL) measured concentrations using Liquid Chromatography/Mass Spectrometry (LC/MS). It cannot be concluded that these different methods resulted in the discrepancy in concentration. To fully reconcile the differences would require a comparison of test methods between the manufacturer and TVMDL, production of new samples, strict control of handling, including temperature and sunlight exposure, and then testing comparisons between the two facilities. Other research in horses tested CBD in plasma and used Liquid Chromatography-tandem mass spectrometry [13,14].

Although the manufacturer tested the product prior to shipment, this study must rely on the independent verification as the levels actually studied for two reasons: (1) the independent verification was on samples taken at the time of the study, and (2) the authors have intimate understanding of the test methods used by TVMDL. Therefore, throughout further discussion of the results of this study, it should be considered that the intake of CBD was 0.13 mg CBD/kg BW on TRTA and 0.12 mg CBD/kg BW on TRTB.

### 4.2. Intake and Performance Characteristics

The current study did not find a difference in the intake of concentrate across treatments, which was expected as horses typically find vegetable oils such as canola oil highly palatable [31]. Similarly, there was no effect of CBD oil supplementation on BW or BCS. A study supplementing 0.5 or 1.5 mg/kg CBD over six weeks found that horses maintained normal appetite, and 10 out of 12 horses in the study gained weight; however, BCS was not reported [14]. A study of CBD supplementation in dogs and cats did not indicate a change in BW after 12 weeks using a dose of 2 mg/kg twice daily [7]. Similarly, Vaughn et al. [32] provided increasing dosages from 1.75 mg/kg to 61.75 mg/kg over a 30 d period without a change in BW, and McGrath et al. [6] administered 2.5 mg/kg twice daily for 12 weeks without a change in BW.

### 4.3. Blood Chemistry Parameters, Enzyme Activity, and Drugs of Abuse Screening

After 28 days of supplementation, levels of serum Ca were within physiologically normal range (11.2 to 13.0 mg/dL, TVMDL) but increased with increasing CBD supplementation. Cannabinoid Type 1 Receptor (CB1R) and Cannabinoid Type 2 Receptor (CB2R) are found in numerous tissues, including the kidneys, as a result of CBD supplementation. Studies suggest that stimulation of these receptors in the kidneys could have positive or negative effects. Cannabinoid Type 2 Receptors interact with heterotrimeric G-proteins consisting of Gαi subunits. The range of CB1R and CB2R response on cell signaling pathways is not fully known.

Cannabinoid Type 1 Receptors have been shown to associate with other Gα subunits to produce a variety of effects, including the increase of intracellular Ca levels [10]. Increased Ca levels beyond normal ranges may indicate chronic renal failure or other disorders in dogs, cats, and horses [8]. This study did not see Ca levels in blood serum exceed normal levels with CBD supplementation, which is similar to previous studies in which values remained within normal limits [5,7,12,32]. However, in a different study, horses given 0.5 mg/kg or 1.5 mg/kg of CBD suspended in sunflower lecithin oil exhibited mild hypocalcemia (10.0–11.4 mg/dL over a 6-week trial) [14].

In contrast to Ca, creatinine levels tended to decrease (*p* = 0.06) with increasing levels of CBD supplementation (CTRL = 1.41 mg/dL, TRTB = 1.34 mg/dL, TRTA = 1.31 mg/dL). Creatinine is a waste output produced by the muscle [11]. The muscle uses a protein known as creatine to generate energy for contractions. Creatinine levels reflect muscle mass and, therefore, are largely constant from day to day. Normal levels of creatinine in horses are 0.9 to 1.7 mg/dL (TVMDL). The kidneys filter creatinine from the blood into the urine. As the muscles are producing creatinine at a fairly constant rate, the removal process is also fairly constant. An increase in creatinine level in the blood is a good indicator of kidney stress. A reduction is indicative of improved kidney function [11]. The current study observed a reduction in creatinine levels with increasing CBD treatment level. A reduction of creatinine can be produced by improving kidney health or function. Increasing fiber in the diet is an example of another means of reducing creatinine [11]. A similar study saw the same reduction in creatinine with CBD supplementation levels of 0.09 mg/kg BW and 0.18 mg/kg BW, yielding 1.41 mg/dL and 1.22 mg/dL, respectively [12].

The remaining blood chemistry parameters, including blood urea nitrogen, total protein, albumin, glucose, phosphorous, sodium, potassium, Na:K, and Cl, were not influenced by dietary treatment. Draeger et al. [12] reported a significant decrease in albumin when comparing horses supplemented with 50 mg compared to 250 mg. The current study did not observe a similar decrease in albumin levels. Furthermore, previous work carried out in dogs and cats has demonstrated similar findings, in which no other changes were observed in blood chemistry values [5,6,7,17,32].

In general, a rise in liver enzymes is concerning due to the many metabolic functions involving the liver. Observation of liver enzymes requires some level of elevated pattern recognition. Alanine aminotransferase and AST will increase together if there is hepatocellular damage. Alkaline phosphatase and GGT will increase together if there is cholestasis. Some liver diseases, such as cholangitis or phenobarbital hepatopathy, can display a mixed pattern [9]. Gamma-glutamyl transferase demonstrated a quadratic dosage response, as TRTB was greater than CTRL. The values were within normal levels of 8 to 33 U/L, and did not indicate any negative effects on the liver in the current study, but it has been demonstrated in other studies carried out in dogs, cats, and horses that elevated liver values indicate potential harmful effects to the liver. Yocum and associates [14] found elevated liver enzymes (gamma-glutamyl transferase, aspartate aminotransferase, and sorbitol dehydrogenase) in eight of twelve horses used in their trial. Gamma-glutamyl transferase is a marker of intrahepatic or extrahepatic cholestasis, and it is associated with the cell membranes of hepatocytes of the bile canaliculi and ducts. It is also associated with periportal hepatocytes. Cannabidiol can cause hepatotoxicity, resulting in elevated GGT [4,9]. In general, gamma-glutamyl transferase is a particularly useful indicator in horses and ruminants for long-term liver damage [8]. The remaining liver enzymes (alkaline phosphatase, creatine kinase, aspartate aminotransferase glutamate dehydrogenase), as well as total bilirubin, bilirubin direct, albumin: globulin, and globulins were not influenced by dietary treatment in this trial. One study by Draeger et al. [12] also examined alkaline phosphatase, and also did not see a difference depending on CBD supplementation. However, in the trial by Yocum et al. [14], an increase in aspartate aminotransferase was observed. Further studies should investigate the effects of CBD on liver function in horses.

Numerous competitive equine associations prohibit the use of CBD and cannabidiolic acid, and their presence would result in disqualification of the horse and competitor. Therefore, this study included a drugs of abuse screening in order to evaluate if dietary treatment influenced detectable substances in the blood. No drugs of abuse (including, but not limited to CBD, 7-carboxycannabidiol, 7-Nor-7-carboxycannabidiol, and tetrahydrocannabinol (THC)) were detected in blood on d 0 and the following 28 d of supplementation in all horses, regardless of treatment.

## 5. Conclusions

We observed a substantial difference in CBD content of our supplement from targeted formulation compared to our tested composited sample collected throughout the trial. Treatment A was formulated to deliver 1.50 mg CBD/kg BW but tested at 0.13 mg/kg, and TRTB was formulated to deliver 0.75 mg/kg but tested at 0.12 mg/kg CBD. Additional investigation is needed in order to understand the stability of CBD oil containing supplements under conditions of typical use. If the discrepancy in formulated amount versus tested amount is due to a difference in the method of analysis, then horses in the current study potentially consumed closer to the targeted levels of CBD, and no major health concerns were noted within the study population; however, as previously described, we must operate using the tested values found from the independent lab analysis. Based on previous studies indicating issues related to kidney and liver function at supplementation levels greater than 2 mg/kg, the tested level of CBD supplementation of 0.12 or 0.13 mg/kg would be unlikely to create problems. Furthermore, if the decreased level of CBD in the current study from formulation to testing was due to problems during handling and storage of the composite during the 28 day trial, then this is a very important factor to consider when considering practical applications of CBD oil supplements in the horse industry. Handling of the supplements in this trial were not outside of any typical use of oil-based supplements, with storage at room temperature and within the barn environment for short periods of time. While circulating levels of CBD and other illegal compounds were not detectable in the current study, this may reflect the level of supplementation and further work is needed to evaluate use in horses involved in competitive events. In conclusion, the canola-based CBD oil was well-accepted by mature horses, banned substances were not detectible in blood, and blood chemistry parameters were not adversely affected as a result of supplementation when added to the diet at levels up to 0.13 mg/kg BW. Further research is required to elucidate the safety of CBD supplemented at higher levels.

## Figures and Tables

**Table 1:** Nutrient composition of pelleted concentrate, coastal bermudagrass (*Cynodon dactylon*) hay, and coastal bermudagrass pasture.

Item ^1^	Concentrate ^2^	Hay	Pasture
Nutrient, % DM			
Crude Protein, %	18.55	6.18	12.87
Starch, %	17.99	2.16	1.49
Crude Fat, %	6.09	1.60	2.33
Acid Detergent Fiber, %	23.23	35.47	37.84
Neutral Detergent Fiber, %	32.34	62.15	62.70
Ca, %	0.82	0.27	0.31
P, %	0.60	0.17	0.29

^1^ Elk River Forage Lab (Elk River, MN); ^2^ 0.75% BW (as-fed) daily of a commercial concentrate (Producer’s Golden Years, Producer’s Cooperative Association, Bryan, TX, USA).

**Table 2:** Blood chemistry parameters (least square means) in serum of mature horses receiving 0.13 mg/kg BW cannabidiol oil (TRTA; n = 10), 0.12 mg/kg BW cannabidiol oil (TRTB; n = 10) or canola oil (CON; n = 10) top-dressed onto a pelleted concentrate.

	Dietary Treatments				*p*-Values ^1^	
Parameter	TRTA	TRTB	CTRL	SEM	Trt	Contrast
Blood Urea Nitrogen, mg/dL	18.16	18.61	18.91	0.61	0.67	0.42
Creatinine, mg/dL	1.31 *	1.34 *^,ǂ^	1.41 ^ǂ^	0.03	0.07	0.06
Total Protein, g/dL	6.38	6.46	6.48	0.08	0.67	0.39
Albumin, g/dL	3.37	3.35	3.32	0.03	0.56	0.32
Glucose, mg/dL	77.14	79.04	80.05	2.10	0.61	0.34
Total bilirubin, mg/dL	1.02	1.06	1.19	0.07	0.16	0.53
Bilirubin direct, mg/dL	0.28	0.27	0.33	0.02	0.17	0.09
Albumin:Globulin	1.09	1.05	1.08	0.02	0.43	0.30
Globulins, g/dL	3.06	3.14	3.08	0.07	0.74	0.53
Calcium, mg/dL	11.67 ^a^	11.42 ^b^	11.37 ^b^	0.06	0.01	0.01
Phosphorous, mg/dL	3.05	3.26	3.27	0.11	0.28	0.12
Sodium, mEq/L	137.19	137.09	137.16	0.32	0.97	0.89
Potassium, mEq/L	3.78	3.87	3.67	0.07	0.13	0.13
Na:K	36.24	35.54	37.08	0.56	0.21	0.82
Chloride, mEq/L	100.02	99.81	99.86	0.39	0.93	0.72

^1^ Trt = main effect of treatment for the 28-d trial; Contrast = linear contrast; ^a,b^ Superscripts denote a difference in dietary treatment (*p* < 0.05); *^,ǂ^ Superscripts denote a tendency in dietary treatment (*p* < 0.10).

**Table 3:** Liver enzyme analytes (least square means) in serum of mature horses receiving 0.13 mg/kg BW cannabidiol oil (TRTA; n = 10), 0.12 mg/kg BW cannabidiol oil (TRTB; n = 10) or canola oil (CTRL; n = 10) top-dressed onto a pelleted concentrate.

	Dietary Treatments				*p*-Values ^1^	
Parameter	TRTA	TRTB	CTRL	SEM	Trt	Contrast
Alkaline phosphatase, U/L	160.77	165.07	156.40	6.17	0.60	0.32
Creatine kinase, U/L	274.22	249.35	223.58	21.52	0.23	0.40
Aspartate aminotransferase	273.56	273.34	280.25	6.37	0.66	0.44
Glutamate Dehydrogenase, U/L	4.48	4.93	4.09	0.63	0.66	0.37
Gamma-glutamyl transferase, U/L	16.58 ^b^	17.38 ^b^	14.86 ^a^	0.77	0.08	0.03

^1^ Trt = main effect of treatment for the 28-d trial; Contrast = quadratic contrast; ^a,b^ Superscripts denote a tendency in dietary treatment (*p* < 0.10).

## Data Availability

Not applicable.
